# Testicular Abscess and Ischemia Secondary to Epididymo-orchitis

**DOI:** 10.7759/cureus.8991

**Published:** 2020-07-03

**Authors:** Brittany Hackett, Zachary Sletten, Rachel E Bridwell

**Affiliations:** 1 Emergency Medicine, San Antonio Military Medical Center, San Antonio, USA; 2 Emergency Medicine, Brooke Army Medical Center, Fort Sam Houston, USA

**Keywords:** orchitis, epididymo-orchitis, testicular infarction, ischemia, epididymitis

## Abstract

Acute testicular infarction requiring emergent surgical intervention is often the result of spermatic cord torsion; infrequently, infarction results from other etiologies. We report a case of epididymo-orchitis complicated by abscess resulting in testicular ischemia, not detected on ultrasonography. A high clinical suspicion of testicular ischemia should be maintained in any presentation of testicular pain and swelling, as recognition could lead to early salvage interventions.

## Introduction

Epididymo-orchitis is a common cause of acute scrotal pain. Approximately 600,000 cases of epididymitis occur annually in the United States. One study reported concomitant orchitis in 58% of men diagnosed with epididymitis [1]. Patients present complaining of testicular or scrotal pain and may have associated fever, fatigue, nausea, and vomiting. Infection is caused by retrograde ascent of pathogens to the epididymis and extends to the testis [2]. The majority of infections are caused by bacteria and occur in a bimodal distribution. In men aged 14 to 35 years, infection is commonly caused by sexually transmitted *Neisseria gonorrhoeae* or *Chlamydia trachomatis*. In men aged 35 years or older, common organisms are *Escherichia coli, Klebsiella pneumoniae, Pseudomonas aeruginosa,* and *Staphylococcus* and *Streptococcus* species [1,2]. Rarely, the infectious process progresses to abscess formation with testicular infarction [2]. We report a case of epididymo-orchitis complicated by abscess resulting in testicular ischemia. 

## Case presentation

**Figure 1:**
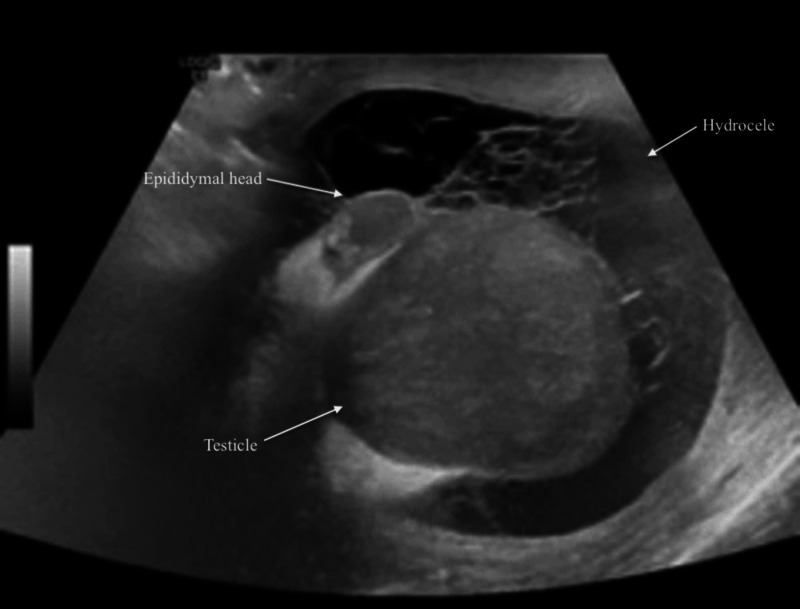
Grayscale ultrasound showing heterogeneous testicle and epididymis, and complex hydrocele.

**Figure 2:**
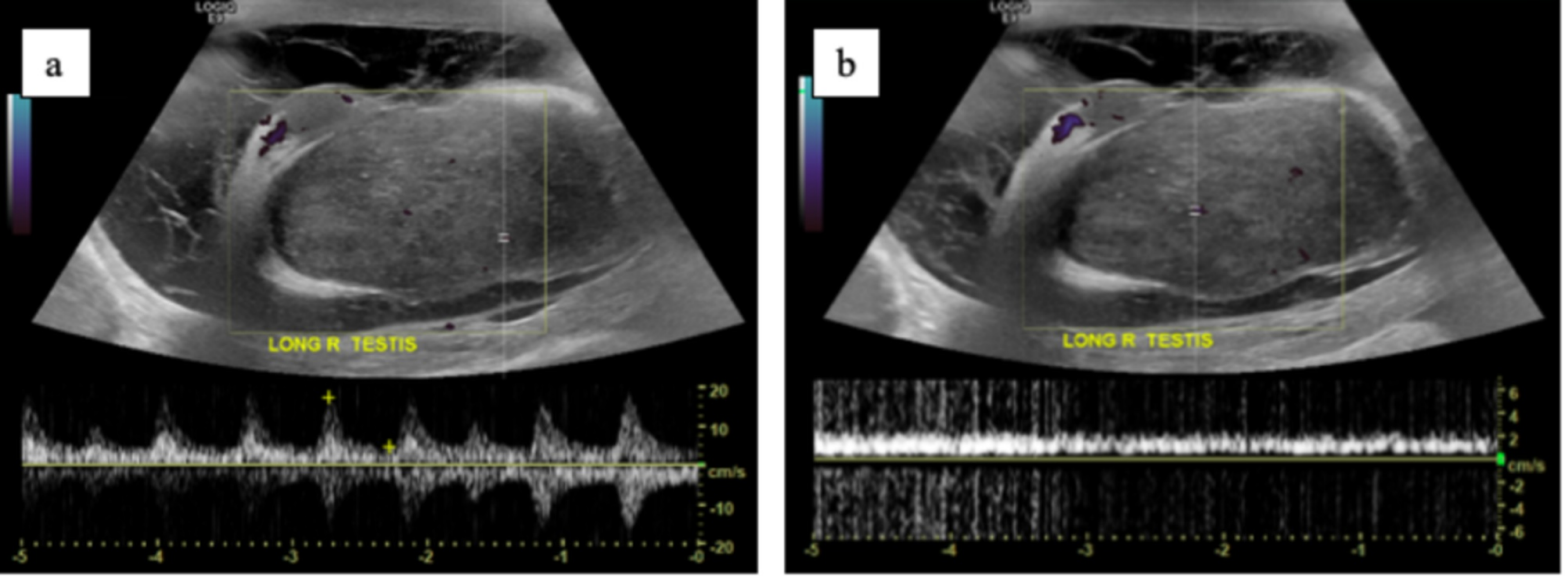
Spectral Doppler ultrasound of right testicle demonstrating normal arterial (a) and venous (b) flow.

A 67-year-old man presented to the emergency department complaining of right scrotal pain and swelling for one week. The patient initially reported swelling of the right scrotum, and pain radiating to the right inguinal region. He also reported chills, fever, nausea, and vomiting, though denied lower urinary tract symptoms. The patient had a history of urinary retention secondary to benign prostatic hypertrophy, rendering him self-catheter dependent for one year. Several weeks prior to presentation, the patient had decreased intermittent self-catheterization to twice daily due to an increase in spontaneous voiding. Additional past medical history included hypertension and chronic kidney disease. Physical examination revealed tachycardia to 108 beats per minute, temperature of 101.7 degrees Fahrenheit, and normal blood pressure. Genital examination was remarkable for a tender and indurated right hemi-scrotum with thinning superficial skin and non-palpable testis due to swelling. Urinalysis revealed the presence of leukocyte esterase, white blood cells (WBCs), and bacteria; serum analysis was notable for leukocytosis (WBC) 21.1 x 10^9^/L with neutrophil predominance (85%). Spectral Doppler ultrasound showed heterogeneous right testis and epididymis, large markedly complex hydrocele (Figure 1), with preserved flow (Figure 2), suggesting epididymo-orchitis. A CT was performed which did not demonstrate abscess or additional pathology. The patient was admitted to the hospital and initiated on piperacillin/tazobactam. 

On hospital day 3, the patient remained febrile with persistent leukocytosis and interval worsening of scrotal edema. Operative exploration found an enlarged and engorged right epididymis and testis with purulence, at which time an orchiectomy was performed. Urine cultures speciated *Klebsiella pneumoniae *and *Escherichia coli*, while intraoperative wound cultures grew *Staphylococcus lugdunensis*. Histology revealed testicular acute and chronic inflammation, abscess formation, and ischemic necrosis. 

Postoperatively, the patient clinically improved with a down-trending leukocytosis and temperature. He was discharged home on cephalexin and was well at his postoperative follow-up.

## Discussion

Testicular abscess formation and ischemia are rare complications of epididymo-orchitis in the setting of appropriate antibiotic therapy [3]. While exact mechanisms remain unknown, proposed mechanisms suggest that compression of the vasculature of the epididymis and testicle effectively creates a compartment syndrome [4,5]. Acute inflammatory changes, exudates, and tissue edema may result in extra luminal compression. Simultaneously, venous congestion with resulting thrombosis and endothelial dysfunction increase pressure, leading to hypoxia. 

Epididymo-orchitis complicated by abscess formation is associated with significant morbidity, predominantly testicular infarction [3,4]. As demonstrated in our case, testicular abscess may not be detected on routine Doppler ultrasound. Alternatively, contrast-enhanced ultrasound (CEUS) can aid in identifying focal pathology. Even in the early stages of abscess formation, images would demonstrate an unenhanced focus surrounded by a hyperenhanced rim. The contrast agent used for CEUS is not nephrotoxic, minimizing impact on those with underlying kidney dysfunction. It can be performed quickly at the patient bedside, making it ideal for emergent evaluation of acute scrotal pain [6-8]. Additionally, in a study of segmental testicular infarction, CEUS was 100% sensitive in detecting infarction [9]. If CEUS is not available and clinical suspicion remains high for ischemia, urgent MRI with T2-weighted images is 100% accurate in detection of necrosis [10]. 

Management of epididymo-orchitis often includes antimicrobial and anti-inflammatory therapy, as the majority of cases resolve without complication. Empiric antibiotics should be based on age and sexual history. If there is suspicion of sexually transmitted pathogens, common in patients less than 35 years, therapy should include cetriaxone 250 mg intramuscularly and doxycycline 100 mg orally twice daily for 10 days. Alternatively, azithromycin 1 g orally can be substituted for doxycycline. If infection is thought to be caused by enteric bacteria, common in men older than 35 years, fluoroquinolones (ciprofloxacin, ofloxacin, levofloxacin) are preferred [1,2]. 

## Conclusions

We describe a case of acute epididymo-orchitis that progressed to abscess formation and ischemia, and ultimately required surgical exploration and orchiectomy. While the exact mechanism of ischemia is still under investigation, elevated compartment pressures may play a role in circulatory deprivation. In the setting of minimal to no clinical improvement after the initiation of antibiotics, CEUS may increase early detection of abscess or ischemia, leading to optimization of therapy. 
